# Transesophageal echocardiography in patients with cryptogenic cerebral ischemia

**DOI:** 10.1186/1476-7120-7-15

**Published:** 2009-03-28

**Authors:** Fabian Knebel, Florian Masuhr, Wolfram von Hausen, Torsten Walde, Henryk Dreger, Vanessa Raab, Mahsun Yuerek, Gert Baumann, Adrian C Borges

**Affiliations:** 1 Charité Universitatsmedizin Berlin, Campus Mitte, Medizinische Klinik und Poliklinik mit Schwerpunkt Kardiologie und Angiologie, Berlin, Germany; 2 Charité Universitatsmedizin Berlin, Campus Mitte, Department of Neurology, Berlin, Germany; 3 Bundeswehrkrankenhaus Berlin, Abteilung 1, Innere Medizin, Scharnhorststrasse 13, 10115 Berlin, Germany; 4 Paritätisches Krankenhaus Lichtenberg, Fanningerstraße 32, 10365 Berlin, Germany; 5 Städtisches Klinikum München Schwabing, Kölner Platz 1, 80804 München, Germany; 6 State University of New York Downstate Medical Center, Children's Hospital at Downstate 450 Clarkson Avenue, Box 49, 11203 Brooklyn, New York, USA

## Abstract

**Background:**

In about one third of all patients with cerebral ischemia, no definite cause can be identified (cryptogenic stroke). In many patients with initially suspected cryptogenic stroke, however, a cardiogenic etiology can eventually be determined. Hence, the aim of this study was to describe the prevalence of abnormal echocardiographic findings in a large number of these patients.

**Method:**

Patients with cryptogenic cerebral ischemia (ischemic stroke, IS, and transient ischemic attack, TIA) were included. The initial work-up included a neurological examination, EEG, cCT, cMRT, 12-lead ECG, Holter-ECG, Doppler ultrasound of the extracranial arteries, and transthoracic echocardiography. A multiplane transeophageal echocardiography (TEE, including i.v. contrast medium application [Echovist], Valsalva maneuver) was performed in all patients

**Results:**

702 consecutive patients (380 male, 383 IS, 319 TIA, age 18–90 years) were included. In 52.6% of all patients, TEE examination revealed relevant findings. Overall, the most common findings in all patients were: patent foramen ovale (21.7%), previously undiagnosed valvular disease (15.8%), aortic plaques, aortic valve sclerosis, atrial septal aneurysms, regional myocardial dyskinesia, dilated left atrium and atrial septal defects. Older patients (> 55 years, n = 291) and patients with IS had more relevant echocardiographic findings than younger patients or patients with TIA, respectively (p = 0.002, p = 0.003). The prevalence rates of PFO or ASD were higher in younger patients (PFO: 26.8% vs. 18.0%, p = 0.005, ASD: 9.6% vs. 4.9%, p = 0.014).

**Conclusion:**

A TEE examination in cryptogenic stroke reveals contributing cardiogenic factors in about half of all patients. Younger patients had a higher prevalence of PFO, whereas older patients had more frequently atherosclerotic findings. Therefore, TEE examinations seem indicated in all patients with cryptogenic stroke – irrespective of age – because of specific therapeutic consequences.

## Background

Cerebral ischemia is among the most common causes of hospitalization, morbidity and mortality in western civilizations. Stroke databases suggest that despite intensive evaluation, approximately 40% of all patients suffering ischemic strokes have no clearly identifiable cause and 15–20% of the ischemic events occur in younger patients (<55 years). In the literature, however, there is no consensus on the percentage of ischemic strokes caused by cardioembolic events [1].

Interatrial communications (ASD, PFO with and without atrial septal aneurysms [ASA]) can lead to paradoxical stroke and increase the risk for recurrent thrombembolic cerebral events. [2-4].

In an autopsy study, the overall prevalence of a PFO was 27.3%. [5] A meta-analysis has shown that a PFO is more common in patients younger than < 55 years with suspected cryptogenic stroke than in healthy controls [6]. Hence, a PFO is considered as a risk factor for ischemic cerebral events in younger patients. In older patients (i.e. > 55 years), the association between strokes and prevalence of a PFO is much weaker [7].

In clinical practice, it is not easy to verify paradoxical embolism. In current guidelines, there is no consensus on the clinical relevance of a PFO in cryptogenic stroke and on the indication for the closure of a PFO after a stroke [8].

The aim of this study was to determine the prevalence of echocardiographic findings by TEE in a large number of patients with cryptogenic cerebral ischemia with a focus on pre-defined subgroups (age, sex, stroke vs. TIA).

## Methods

In this single center retrospective study, 702 consecutive patients with acute cerebral ischemia were examined by TEE (1996–2001). All patients underwent neurological clinical examination, electroencephalogram, cCT or cMRT, and laboratory tests. All patients had a 12-lead-ECG, Holter-ECG, extracranial color-coded sonography, transthoracic echocardiography with no explanations for cerebral ischemia.

Exclusion criteria were: hemodynamically relevant stenoses of the intra- and extracranial arteries, cerebral hemorrhage, atrial fibrillation, migraine, epilepsy, intracranial tumors, acute myocardial infarction in the previous four weeks, previously diagnosed chronic heart failure and left ventricular aneurysms and less than 18 years of age.

Ischemic stroke was defined as a cerebrovascular event with symptoms lasting longer than 24 hours. Diagnosis of ischemic stroke or TIA was confirmed by a study neurologist based on clinical syndrome and results of diagnostic tests, including CT/MRI scans.

Transesophageal echocardiography was performed with a multiplane probe (5–7 MHz) on a Vivid 5 (TEE probe MPTE 5MHz 6A, GE Vingmed, Horton, Norway) and a HP Sonos 5500 (TEE probe HP 21369A; Hewlett-Packard, Paolo Alto, California, USA).

Before transesophageal echocardiography, intraoral xylocaine spray and intravenous midazolam were administered according to the requirements of the patient. The heart rhythm was monitored by ECG during the examination.

Each TEE examination included the standard views and measurement of blood velocities in the left atrial appendage, intravenous administration of contrast agent and Valsalva manoeuvre to exclude interatrial communications (according to [9]).

The images were stored digitally and analyzed off-line by EchoPac PC Dimension (GE Vingmed, Horton Norway) and additionally on video. Echovist 300 was used as contrast medium (Galactose; Schering, Berlin, Germany). Echovist is approved for the diagnosis of intra-atrial communications.

Written consent was obtained from each patient for the TEE, and the ethics committee of the Charité University Hospital approved the protocol.

### Statistics

Statistics were calculated by Statgraphics plus (Version 6, Herndon, Virginia USA). Results are expressed as mean (± standard deviation). Comparisons of parametric variables between the responders and the non-responders were calculated by paired Student's t-test. The comparison of echocardiographic parameters between groups was calculated by unpaired Whitney-Mann test. Dichotomized data were analyzed by the Chi^2^-test. The level of significance was p ≤ 0.05.

## Results

702 consecutive patients were included. 380 (54.1%) were male, 322 (45.9%) female. 383 (54.6%) of the patients had an IS and 319 (45.4%) had a TIA. 411 patients were younger than 55 years (58.5%) and 291 patients were older than 55 years (41.5%).

The stroke subgroup was significantly older and had more frequently pathological TEE findings than the patients suffering from a TIA. (Table 1).

**Table 1:** Patient characteristics (median, ± SD, n [%])

	**All (n = 702)**	**Ischemic stroke (n = 383, 54.6%)**	**TIA (n = 319, 45.4%))**	**p**
**Age**	57.1 (± 15.7)	61.5 (± 12.3)	51.3 (± 16.2)	**<0.001**

**Male Sex (%)**	380 (54.1%)	222 (57.9%)	158 (49.5%)	**0.043**

**Diabetes mellitus**	13.0%	21.2%	7.0%	0.17

**Arterial hypertension**	39.5%	59.4%	25.6%	0.09

**Hyperlipidemia**	35.5%	50.0%	25.6%	0.07

**Smoker**	26.0%	27.6%	25.6%	0.82

**Coronary artery disease**	14.3%	21.2%	9.3%	0.31

**Any pathological echo finding**	369 (52.6%)	225 (58.7%)	144 (45.1%)	**0.02**

**Number of pathological findings if any**	2.24	2.43	1.92	**0.003**

The prevalence of abnormal echocardiographic findings is listed in (Table 2). Neither the presence of a PFO (p = 0.053), nor of an ASD (p = 0.65) or an atrial septal aneurysm (p = 0.21) was significantly different in the IS and TIA groups. However, findings attributable to atherosclerosis (valvular abnormalities including mitral valve calcification, aortic calcification, aortic valve sclerosis, aortic plaque) were significantly more frequent in the ischemic stroke group (Table 3).

**Table 2:** Echocardiographic findings in all patients.

**Finding**	**n**	**%**
*Any echocardiographic finding*	*369*	*52.6*

		

Patent Foramen ovale (PFO)	152	21.7

Valvular abnormalities	111	15.8

Aortic plaques	102	14.5

Aortic valve sclerosis	66	9.4

Atrial septal aneurysm	51	7.3

regional myocardial dyskinesia in > 2 segments	50	7.1

Left atrial dilatation	47	6.7

Atrial septal defect	28	4.0

Spontaneous echo contrast (SEC)	18	2.6

Mitral valve prolapse	15	2.1

Valvular vegetations	14	2.0

Aortic valve strands	14	2.0

Intracardial thrombi	13	1.9

mitral valve annulus calcification	10	1.4

Chiari networkt	6	0.9

Aortic valve stenosis	5	0.7

Mitral valve strands	5	0.7

Aortic thrombi	4	0.6

Aortic aneurysm	3	0.4

Prosthetic valve	3	0.43

Left ventricular dilatation	3	0.43

Intracardiac tumor	1	0.14

Mitral valve stenosis	1	0.14

**Table 3:** The 10 most frequent findings in the stroke and TIA subgroups

**Rank**	**Ischemic stroke (n = 383)**	**TIA (n = 319)**	**P (Chi2 Pearson)**
1	PFO (94; 24.5%)	PFO (58; 18.2%)	**0.053**

2	Valvular abnormalities (73; 19.1%)	Valvular abnormalities (38; 11.9%)	**0.014**

3	Aortic calcification (73; 19.1%)	Aortic calcification (32; 10.0%)	**0.001**

4	Aortic plaque (71; 18.5%)	Aortic plaque (31; 9.7%)	**0.002**

5	Aoric valve aclerosis (45; 11.7%)	Aoric valve aclerosis (21; 6.6%)	**0.029**

6	Atrial septal aneurysm (36; 9.4%)	Left atrial dilatation (20; 6.3%)	LA dilatation: 0.807

7	regional myocardial dyskinesia in > 2 segments (33; 8.6%)	regional myocardial dyskinesia in > 2 segments (17; 5.3%)	0.131

8	Left atrial dilatation (27; 7.0%)	Atrial septal aneurysm (15; 4.7%)	ASA 0.213

9	ASD (17; 4.4%)	ASD (11; 3.4%)	0.65

10	SEC(16; 4.2%)	Mitral valve prolapse (6; 1.9%)	

**Figure 1:**
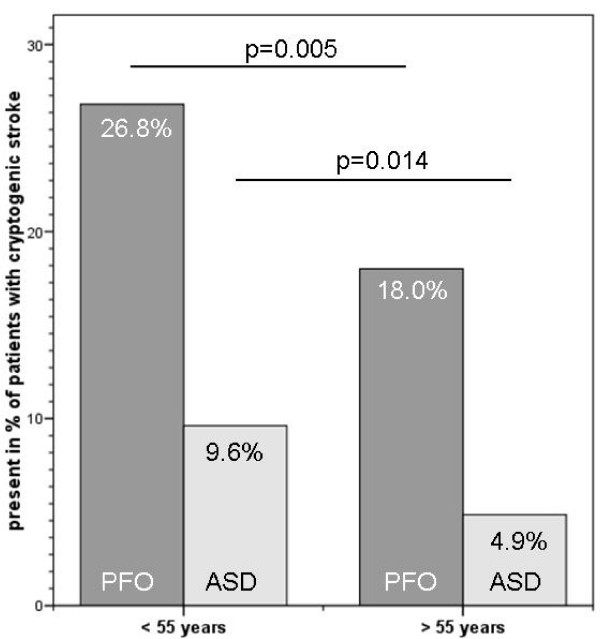
**Presence of PFO and ASD in the patients with cryptogenic stroke (n = 291 < 55 years; n = 411 > 55 years)**.

**Figure 2:**
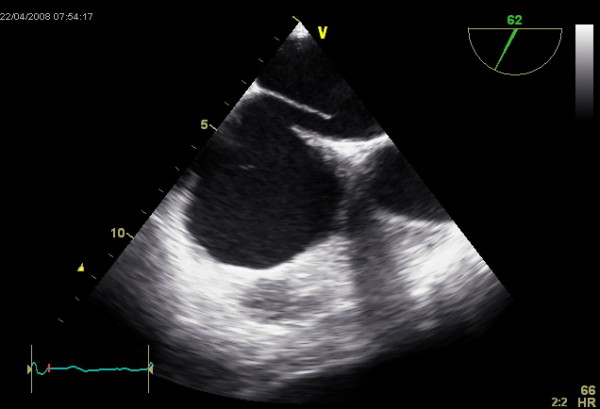
**TEE image showing a PFO with the clearly visible interatrial communication**.

**Figure 3:**
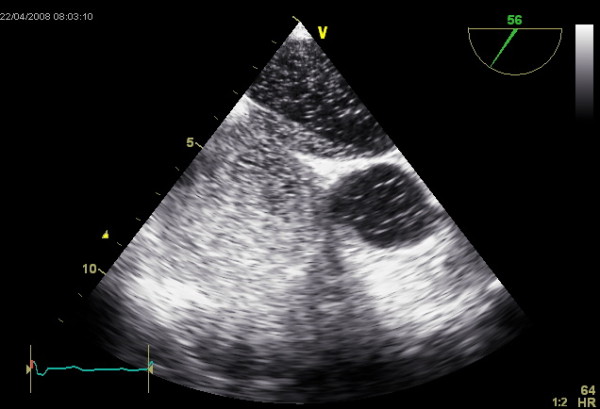
**TEE showing the transfer of EchoVist from the right to the left atrium during Valsalva's manoeuvre**.

**Figure 4:**
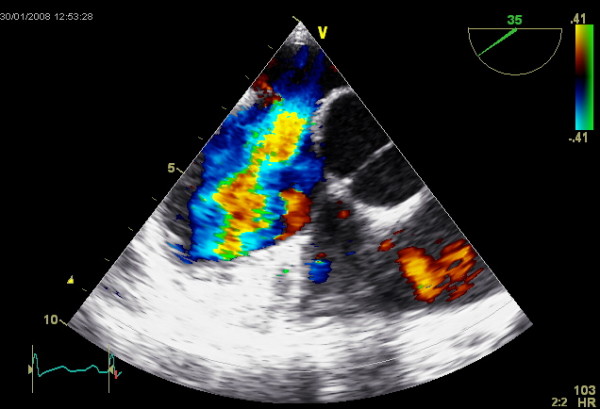
**TEE image showing an ASD and the color coded Dopper visualizing the left-to-right shunt**.

In the older patients (> 55 years, n = 291, according to [2,10]) pathological echocardiographic findings were more frequent (p = 0.0023) than in the younger patients. The prevalence of a PFO was higher in the younger patients (< 55 years: n = 78; 26.8%, > 55 years: n = 75; 18.0%, Pearson's Chi square p = 0.005). The frequency of a ASD was lower in the older patients (< 55 years: n = 28; 9.6%, > 55 years: n = 20; 4.9%, Pearson's Chi square p = 0.014); see Figure 1, for examples see Figure 2, Figure 3 and Figure 4 and Additional file 1, Additional file 2 and Additional file 3

The most frequent combination of two findings was PFO+ASA (2.63% male with IS, 2.71% male with TIA, 2.63% female with TIA, 2.71% female with IS).

## Discussion

This study is to our knowledge the largest TEE study in patients with cryptogenic cerebral ischemia allowing the analysis of the full spectrum of findings and especially the prevalence of a PFO.

In half of all patients, a possible cardiogenic cause was identified by TEE. The most frequent finding was a PFO. The frequency of positive TEE findings that might explain cerebral ischemia is comparable to previous smaller studies [11,12].

Cardiogenic causes of ischemic stroke can be divided into major (annual incidence of embolic events > 1%) and minor risk factors (annual incidence < 1%) [13]. The major risk factors are atrial fibrillation (1–12%/year), intracardiac thrombi (0–35%), atrial myxoma (30–40%), mitral valve stenosis (8–14% in sinus rhythm, 31–65% with concomitant atrial fibrillation), recent myocardial infarction (1–2%), anticoagulated mechanical heart valves (1.5–3%), infective endocarditis (12–40%), dilated cardiomyopathy (4%) and aortic arch atheromatous plaques (4–16% especially if ≥ 4 mm in diameter, [14]). The minor risk factors include mitral valve prolapse (<0.02%) and left ventricular aneurysm (<1%). Rare causes of cardioembolism include Chiari network, Lambl's excrescences [15], and valvular abnormalities [16,17]. Spontaneous echo contrast (SEC) is seen in areas of blood stasis with a slowly moving, cloud-like swirling pattern of "smoke" or increased echogenicity recorded in TEE. However, there is little data on the precise embolic risk of mitral valve annular calcification, spontaneous echo contrast, atrial septal aneurysm, and calcific aortic stenosis [13].

A substantial subset of the cardiogenic factors diagnosed by TEE could have been found with TTE (in combination with trans-cranial Doppler) as well. However, there are no prospective studies showing equivalent diagnostic accuracy of TTE compared to TEE in this setting. Despite the semi-invasivity of TEE, it is still the gold standard for the detection of inter-atrial communications.

### TIA vs. stroke

Patients with TIA had less frequently a pathological finding in the TEE. This could be due to the fact that the initially suspected diagnosis "TIA" has a lower specificity with a broader range of other possible non-vascular etiologies such as migraine or focal seizures.

### Age

We found a higher prevalence of PFO and ASD in the younger patients (< 55 years). In contrast, Handke [10] reported that PFO are equally distributed in all age groups of patients with cryptogenic stroke. Despite these differences, relevant causes for cerebral ischemia can be identified in all age-groups. Therefore, a restriction of a TEE examination to younger patients with TIA/stroke is not supported by our data.

The current data does not allow a clear recommendation for PFO closure in patients with cryptogenic stroke. Further prospective studies are needed to decide on the clinical advantage of PFO closure, especially in patients > 55 years of age.

### Cardioembolic factors – consequences

The TEE findings can have specific therapeutic consequences in all age groups (see table 4). Specifically, as the therapeutic consequence of LA-appendage thrombus is oral anticoagulation, its presence should be ruled out before further treatment is limited to platelet aggregation inhibition alone [13,18].

**Table 4:** TEE findings in patients with ischemic stroke and their possible therapeutic consequences.

**TEE finding**	**Possible therapeutic consequence**
PFO and ASD	ASS, anticoagulation, operative or interventional device closure

aortic plaques	ASS, statin therapy

reduced LVEF	oral anticoagulation, ASS, coronary angiography, heart failure therapy

left atrial dilatation	further cardiological work-up

Spontaneous echo contrast	search for intermittent atrial fibrillation, oral anticoagulation

left atrial thrombus	anticoagulation, operation

thoracic aneurysm of the aorta	echo control, operation

mitral valve prolapse	antiarrhythmic therapy, echo follow-up, anticoagulation

aortic/mitral valve stenosis	timing of valve replacement

LA-appendage thrombus	oral anticoagulation

### PFO-ASA

The association of ischemic stroke and PFO is still controversial: A recent large study in a multiethnic population did not confirm an association of PFO and the risk for an ischemic stroke [19]. However, two studies [20,21]have seen a clear association of PFO and ASA in ischemic stroke.

The diagnosis of a PFO or ASA depends on experienced echocardiographers and has – even among experienced examiners – a high inter- and intra-observer variability [3]. Clear diagnostic criteria for intra-atrial defects could reduce the variability [9].

Surgical [22] or percutaneous closure of a PFO is a therapeutic strategy with somewhat inconsistent results [23,24]. The percutaneous closure of a PFO after recurrent cerebral ischemia is a safe procedure and leads to a reduction of recurrence rates in the long-term follow-up. Only in a small number of patients, there was a residual shunt with subsequent TIA within the first six months after closure [25]. The recurrent ischemic events in these studies were attributed to a residual post-procedural shunt. Hence, improvement of occluder devices may further reduce the recurrence rates after PFO closure.

Conservative strategies (i.e. antithrombotic therapy) in patients with a PFO are also associated with comparable recurrence rates [26]. However, the risks of antithrombotic therapy (platelet inhibition, oral anticoagulation) include bleeding and low compliance rates. A randomized study (PICSS) did not reveal a significant difference in recurrence rates in medically treated patients with or without a PFO [4]. Especially in younger patients on medical therapy, the presence of a PFO did not increase the risk of stroke recurrence [27].

In addition to cardiogenic factors, recently, genetic polymorphisms of a variety of genes have been associated with cryptogenic stroke. However, the degree of association and the diagnostic relevance of these genetic polymorphisms is currently not clear [28].

On the background of our study, we recommend the following diagnostic algorithm for patients with cryptogenic stroke, which as applied in this study: clinical examination, electroencephalogram, cCT or cMRT, 12-lead-ECG, Holter-ECG, extracranial color-coded sonography, transcranial Doppler, transthoracic echocardiography. Only if these examinations do not reveal a cause for the stroke, a TEE seems indicated.

## Conclusion

In conclusion, in this large study in patients with cryptogenic stroke, a TEE reveals in about half of the patients cardiogenic factors that might explain the stroke. Especially, the prevalence of a PFO is higher in the younger patients (< 55 years of age). Therefore, a TEE seems indicated in all patients with cryptogenic stroke – irrespective of age – because of specific therapeutic consequences.

## Limitations

This study was observational. The quality of observational studies seems to be comparable to randomized controlled trials [29,30]. There was no follow-up of the patients concerning recurrence of cerebral ischemia or mortality.

We did not analyze the prevalence of abnormal TEE findings in single-vessel and lacunar strokes in comparison to multi-vessel strokes. The data of our study is historical (1996–2001). Currently, the indication for a TEE in cryptogenic stroke is more selective.

## Competing interests

The authors declare that they have no competing interests.

## Author's contributions

FK and FM equally contributed to the study. FK, FM, WVH, ACB have designed the study, and have performed the examinations. FK, ACB, FM analyzed the data and have written the manuscript. MY, VR, TW, HD performed the TEE examination, collected and interpreted the acquired data. GB has supervised the study and contributed by revising the manuscript critically.

## Supplementary Material

Additional file 1 TEE showing a PFO in 62°. Click here for file

Additional file 2 TEE showing inter-atrial transfer of EchoVist from the right atrium to the left atrium during Valsalva maneuver. Click here for file

Additional file 3 TEE showing a large ASD. Click here for file
